# Effects of Different Delocalized π-Conjugated Systems Towards the TiO_2_-Based Hybrid Photocatalysts

**DOI:** 10.3389/fchem.2021.700380

**Published:** 2021-07-27

**Authors:** Weibo Zhang, Pinghua Chen, Jun Liu, NanNan Huang, Chenglian Feng, Daishe Wu, Yingchen Bai

**Affiliations:** ^1^ Key Laboratory of Poyang Lake Environment and Resource Utilization, Ministry of Education, School of Resources Environmental and Chemical Engineering, Nanchang University, Nanchang, China; ^2^ Key Laboratory of Jiangxi Province for Persistent Pollutants Control and Resources Recycle, Nanchang, China; ^3^ College of Environmental and Chemical Engineering, Nanchang Hangkong University, Nanchang, China; ^4^ State Key Laboratory of Environmental Criteria and Risk Assessment, Chinese Research Academy of Environmental Sciences, Beijing, China

**Keywords:** π-conjugated systems, photocatalyst, phenanthroline, derivatives of phenanthroline, TiO_2_

## Abstract

Modulating the structure of a photocatalyst at the molecular level can improve the photocatalytic efficiency and provides a guide for the synthesis of highly qualified photocatalysts. In this study, TiO_2_ was modified by various organic compounds to form different TiO_2_-based hybrid photocatalysts. 1,10-Phenanthroline (Phen) is an organic material with delocalized π-conjugated systems. It was used to modify TiO_2_ to form the hybrid photocatalyst Phen/TiO_2_. Furthermore, 1,10-phenanthrolin-5-amine (Phen-NH_2_) and 1,10-phenanthroline-5-nitro (Phen-NO_2_) were also used to modify TiO_2_ to form NH_2_-Phen/TiO_2_ and NO_2_-Phen/TiO_2_, respectively. The samples of TiO_2_, Phen/TiO_2_, NO_2_-Phen/TiO_2_, and NH_2_-Phen/TiO_2_ were carefully characterized, and their photocatalytic performance was compared. The results indicated that the photocatalytic efficiency followed the order of NH_2_-Phen/TiO_2_ > NO_2_-Phen/TiO_2_ > Phen/TiO_2_ > TiO_2_. It could be found that modifying TiO_2_ with different organic compounds containing delocalized π-conjugated systems could enhance the photocatalytic ability; furthermore, the level of this enhancement could be modulated by different delocalized π-conjugated systems.

## Introduction

Wastewater is a serious environmental problem as it contains a large number of hazardous organic compounds, such as polycyclic aromatic hydrocarbons, pharmaceuticals (PhACs), and organic dyes (Grzechulska-Damszel et al., 2009; Kim et al., 2013; Li et al., 2018; Murgolo et al., 2021). Therefore, there is an urgent need to find a technology to deal with these pollution problems. Photocatalysis is a green and efficient technique, which has become significant in the field of environmental science because it can utilize the renewable solar energy for the removal of organic pollutants in wastewater (Chen et al., 2010; Chong et al., 2010; Kubacka et al., 2012; Yang et al., 2018). Titanium dioxide (TiO_2_) is one of the most important photocatalysts due to its environment-friendly nature, non-toxicity, chemical stability, and low cost (Liu et al., 2009; Awfa et al., 2018; Gopinath et al., 2020). However, TiO_2_ still has two major disadvantages: one is that pure TiO_2_ has a large band gap (e.g., = 3.0–3.2 eV), which means TiO_2_ can absorb only the ultraviolet light in the photocatalytic reaction, and the other is its high recombination rate of photoinduced electron–hole pairs (Cottineau et al., 2014; Pang et al., 2016; Murgolo et al., 2021). Thus, there is an urgent need to improve the quantum efficiency and light response range of titanium dioxide by modification.

Recently, surface decoration, such as surface coating with metallic oxide, dye grafting on a TiO_2_ surface, and introducing an organic π-conjugated system into the surface, has been developed for reducing the electron–hole recombination rate and increasing the range or intensity of light absorbed by TiO_2_ (Kaur and Singh, 2007; Carbuloni et al., 2020; Xu et al., 2021). The main reason behind the development of surface decoration is that some special structures such as heterojunctions or π-conjugated systems are formed between TiO_2_ and the foreign substance, which can alter the interfacial charge-transfer (ICT) dynamics between TiO_2_ and its surface materials (Ardo and Meyer, 2009; Jono et al., 2011; Fujisawa et al., 2016). The interfacial charge-transfer (ICT) involved in the interface interaction between wide band gap inorganic semiconductors such as TiO_2_ and organic materials have attracted increasing attention due to being beneficial to absorption of visible light and direct electron-injection to TiO_2_ (Ramakrishna and Ghosh, 2002; Verma and Ghosh, 2014; Fujisawa et al., 2017). Furthermore, some new organic molecules that contain a donor–π–acceptor (D-π-A) structure extend the intramolecular charge-transfer time and distance, providing more opportunities for the synthesis of highly efficient photochemical materials (Edvinsson et al., 2007; Tian et al., 2008; Cai et al., 2011). The π-conjugated system modified by functionalized groups such as electron-donating/withdrawing groups has been developed for modifying the electronic structure of organic compounds, which affect the performance of catalysts (Shibano et al., 2007; Verma and Ghosh, 2014; Margalias et al., 2015; Xie et al., 2020). Therefore, it is necessary to analyze the relationship between the organic material that is modified by functionalized groups and the activity of TiO_2_ and light, which help synthesize highly efficient TiO_2_-based catalysts to achieve efficient photocatalytic degradation of organic pollutants in water.

1,10-Phenanthroline (Phen) and its derivatives have a wide range of application in areas such as synthesis of conjugated organic materials due to their high charge-transfer mobility and good electro/photoactive properties (Wei et al., 2018; Çakar, 2019). In this work, 1,10-phenanthroline (Phen), 1,10-phenanthrolin-5-amine (Phen-NH_2_), and 1,10-phenanthroline-5-nitro (Phen-NO_2_) were used to modify TiO_2_ to form Phen/TiO_2_, NH_2_-Phen/TiO_2_, and NO_2_-Phen/TiO_2_, respectively. The morphology, structure, and photoelectric property of the as-prepared photocatalysts were characterized. Their photocatalytic activity was evaluated by photodegradation of methyl orange (MO) under visible light irradiation. The effects of various conjugated systems on their visible light photocatalytic activity were also investigated. This study provides a guide for the synthesis of highly efficient TiO_2_-based catalysts to achieve efficient photocatalytic degradation of organic pollutants in water.

## Experiment

### Catalyst Preparation

#### Synthesis of Phen-NO_2_ and Phen-NH_2_

**FIGURE 1:**
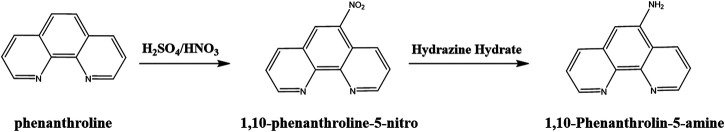
The synthetic routes and structures of phenanthroline, Phen-NO_2_, and Phen-NH_2_.

All starting materials were purchased in an analytically pure form from Aladdin Chemical Reagent Co., Ltd. and utilized without further purification. Phen-NO_2_ and Phen-NH_2_ were prepared following the method in our previous work (Jiang et al., 2016). Typically, to a solution of H_2_SO_4_, phenanthroline was added at room temperature and then a mixture of H_2_SO_4_ and HNO_3_ (1:1) was slowly added. The resulting mixture was refluxed for 3 h, and Phen-NO_2_ was obtained after recrystallization with ethanol. Phen-NO_2_ was reduced to Phen-NH_2_ with hydrazine hydrate. The synthetic routes and structures of phenanthroline, Phen-NO_2_, and Phen-NH_2_ are shown in Figure 1.

#### Synthesis of NH_2_-Phen/TiO_2_, NO_2_-Phen/TiO_2_, Phen/TiO_2_, and TiO_2_

Typically, 3 ml of titanium tetrabutoxide (TBOB) and 0.44 mmol of Phen-NH_2_ were added into 20 ml of ethanol and stirred for 30 min to form a uniform solution. Then, 50 ml of deionized water was added in drops, and the solution was continuously stirred for another 1 h. Then, the mixture was transferred to a Teflon-lined stainless-steel autoclave and maintained at 180°C for 24 h. Finally, the NH_2_-Phen/TiO_2_ obtained after centrifugation was washed with ethanol and deionized water several times and dried in a vacuum oven at 60°C for 12 h. NO_2_-Phen/TiO_2_ and Phen/TiO_2_ were prepared by the method described above in which Phen-NO_2_ and Phen replaced Phen-NH_2_. Bare TiO_2_ was also prepared by the same method without the addition of organic compounds.

### Characterization

The morphologies of the powders were analyzed by using a scanning electron microscope (SEM) (Japanese JEOL JSM-6360). X-ray diffraction (XRD) was performed by using a D8 X-ray diffractometer. The ultraviolet–visible (UV–Vis) diffuse reflectance spectra (DRS) were obtained by using a UV-Vis NIR spectrometer (Lambda 900). The fluorescence spectrum was measured by using a fluorescence spectrometer (F-7000, Japan). Electrochemical impedance and Mott–Schottky curves were recorded on an electrochemical workstation (CHI660C, Shanghai Chenhua, China) with a standard three-electrode system at room temperature. Photoluminescence spectra (PL) were recorded on an F-7000 fluorescence spectrophotometer (Hitachi, Japan).

### Photocatalytic Experiments

The photocatalytic activity of all as-prepared catalysts was evaluated by the degradation of MO under visible light irradiation. The light source was a 500-W Xe illuminator (PerfectLight, Beijing, China). In each experiment, 70 mg of the catalyst was added into 70 ml of MO solution (10 mg/L) in a clean beaker. Before illumination, the suspension was stirred for 30 min in the dark, resulting in a quick adsorption saturation. Then, the suspension was exposed to visible light irradiation with magnetic stirring. At the given time intervals, the suspension was sampled and the photocatalytic particles were removed from the solution using a membrane filter. The concentration of MO in the solution was measured by its absorption intensity at 464 nm.

## Results and Discussion

**FIGURE 2:**
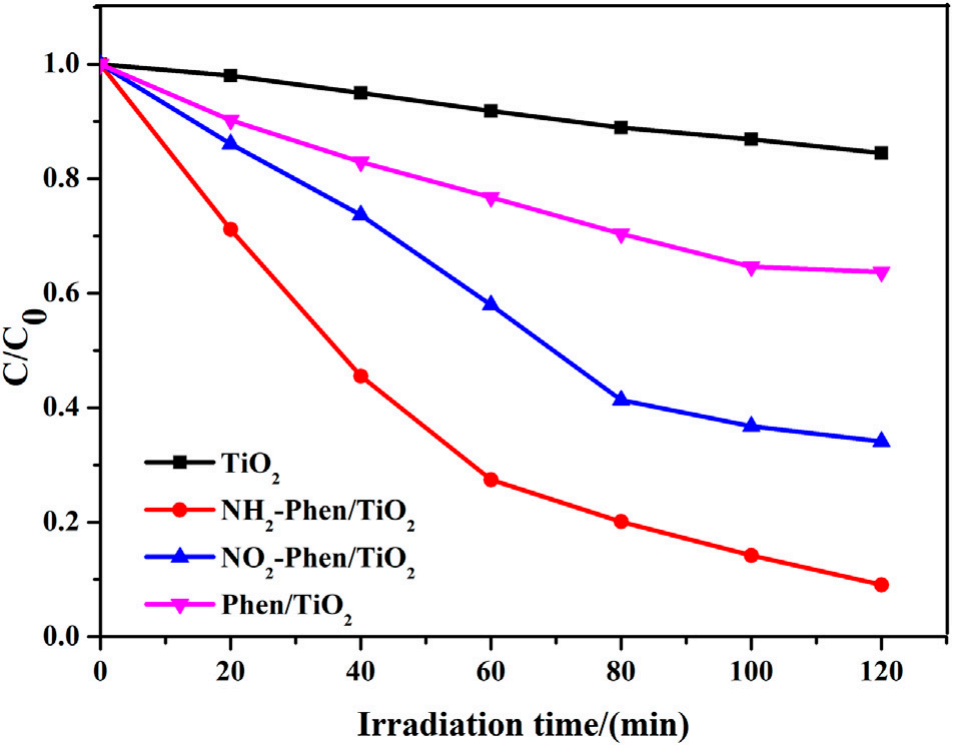
Photoreduction of MO over the as-prepared materials under visible light (70 mg catalysts and 70 ml MO solution with a concentration of 10 mg/L).

The degradation rates of methyl orange (MO) adsorbed on the catalysts under visible light in 120 min are illustrated in Figure 2. NH_2_-Phen/TiO_2_, NO_2_-Phen/TiO_2_, and Phen/TiO_2_ showed higher photocatalytic activity than bare TiO_2_, indicating that Phen, Phen-NH_2_, and Phen-NO_2_ significantly improved the photocatalytic activity in the composites. As shown in Supplementary Figure S1, TiO_2_ has almost no adsorption capacity toward MO. The adsorption capacity of modified TiO_2_ significantly increases and reaches adsorption saturation within 30 min. The final adsorption capacity of NO_2_-Phen/TiO_2_ and NH_2_-Phen/TiO_2_ is almost stable, which is lower than that of Phen/TiO_2_. However, the catalytic efficiency of NO_2_-Phen/TiO_2_ and NH_2_-Phen/TiO_2_ is higher than that of Phen/TiO_2_, indicating that photodegradation is the main reason for MO removal. The catalytic efficiency of NH_2_-Phen/TiO_2_ is the highest (91%), which is nearly 2.5 times and 5.7 times that of Phen/TiO_2_ (36%) and bare TiO_2_ (16%), respectively. NO_2_-Phen/TiO_2_ has the second highest catalytic efficiency (66%), which is nearly 1.8 times and 4.1 times that of Phen/TiO_2_ and bare TiO_2_, respectively. These results showed that the delocalized π-conjugated system with the amino group is more conducive to photodegradation of MO. It is reported that the electron-donating group can induce HOMO-LUMO electronic transitions that cause a change in the dipole moment, which results in effective separation of photogenerated charges (Belviso et al., 2019; Li et al., 2020). Therefore, it is notable that the catalytic efficiency followed the order of NH_2_-Phen/TiO_2_ > NO_2_-Phen/TiO_2_ > Phen/TiO_2_, which indicates that −NH_2_ has a strong electron-donating group than −NO_2_.

**FIGURE 3:**
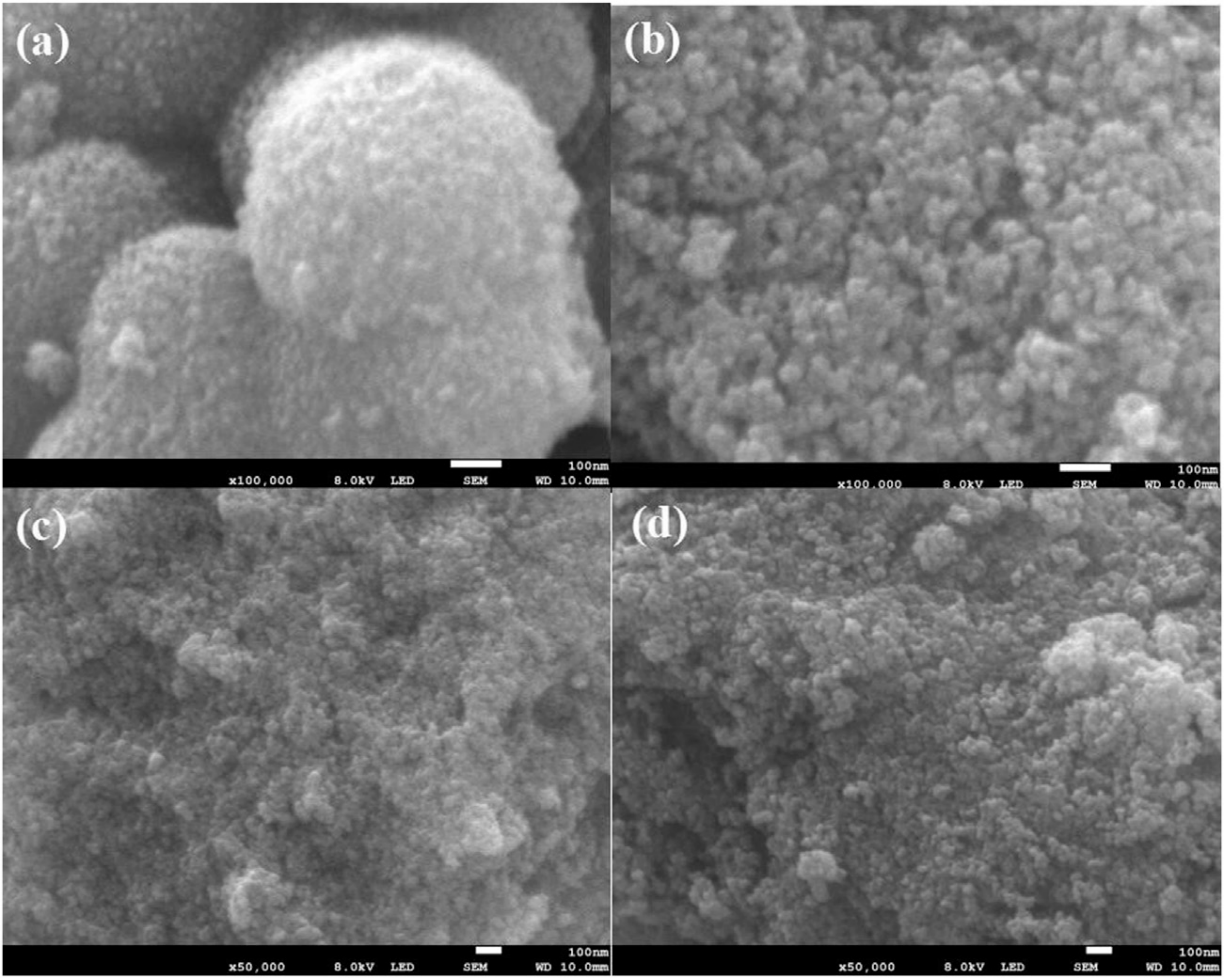
SEM image of TiO_2_ **(A)**, Phen/TiO_2_ **(B)**, NO_2_-Phen/TiO_2_ **(C)**, and NH_2_-Phen/TiO_2_ **(D)**.

Figure 3 shows scanning electron microscopic (SEM) images of NH_2_-Phen/TiO_2_, NO_2_-Phen/TiO_2_, Phen/TiO_2_, and TiO_2_, indicating that the morphologies of NH_2_-Phen/TiO_2_, NO_2_-Phen/TiO_2_, and Phen/TiO_2_ are not significantly different than that of bare TiO_2_. All these samples show similar morphologies with irregular nanoparticles, which means that the preparation methods cannot obviously change the morphologies of TiO_2_ particles. Furthermore, severe agglomeration of the TiO_2_ nanoparticles is also detected, while the modified TiO_2_ nanoparticles have better dispersion, indicating that the modification of TiO_2_ with Phen and its derivatives is beneficial to the dispersion of the nanoparticles. It was reported that the catalytic activity was related to the dispersion of the nanoparticles because a better dispersion could result in a better exposure of active sites (Xun et al., 2020). Therefore, improved dispersion may be another reason for the improved catalytic activity of the modified TiO_2_ catalysts.

**FIGURE 4:**
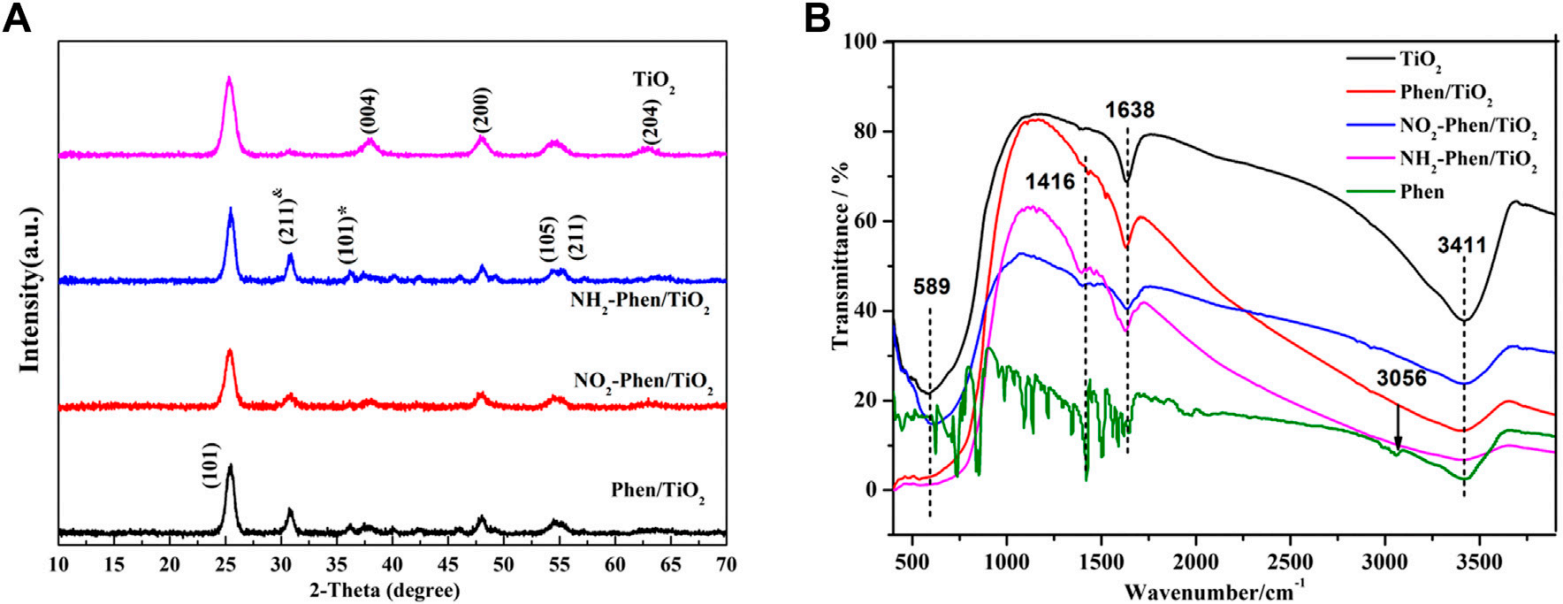
XRD **(A)** and FT-IR patterns **(B)** of TiO_2_, Phen/TiO_2_, NO_2_-Phen/TiO_2_, and NH_2_-Phen/TiO_2_.

X-ray diffraction (XRD) was used to analyze the structure of the catalysts. Figure 4A shows the XRD patterns of NH_2_-Phen/TiO_2_, NO_2_-Phen/TiO_2_, Phen/TiO_2_, and bare TiO_2_. The diffraction peaks of bare TiO_2_ observed at 25.2°, 37.8°, 48.0°, 53.9°, 55.0°, and 62.6° are consistent with anatase TiO_2_ (101), (004), (200), (105), (211), and (204) lattice planes (JCPDS Card No. 21-1272), respectively. While comparing the diffraction peaks of TiO_2_, Phen/TiO_2_, NO_2_-Phen/TiO_2_, and NH_2_-Phen/TiO_2_, all show two new characteristic peaks at 2θ of 30.7° and 36.2°, which correspond to the (211) plane of titanite TiO_2_ and the (101) plane of rutile TiO_2_, respectively. This indicates that the modification of Phen, Phen-NO_2_, and Phen-NH_2_ has caused two new phase structures of brookite TiO_2_ and rutile TiO_2_ appear in NH_2_-Phen/TiO_2_, NO_2_-Phen/TiO_2_, and Phen/TiO_2_ along with the anatase TiO_2_ phase. These results indicate that the modification of Phen and its derivatives is beneficial for forming brookite TiO_2_ and results in a mixed phase of anatase, brookite, and rutile TiO_2_ in the catalysts. The changes in crystalline phases imply the successful modification of Phen and its derivatives. Furthermore, a mixture of different crystalline phases could give rise to a higher photocatalytic activity (Dai et al., 2015; Kandiel et al., 2013). According to the Scherrer formula (Cao et al., 2011), the lattice sizes of TiO_2_, Phen/TiO_2_, NO_2_-Phen/TiO_2_, and NH_2_-Phen/TiO_2_ are 9.35, 11.03, 10.82, and 9.44 nm, respectively.

Infrared characterization was performed to determine the functional groups present in the catalysts. Figure 4B shows the FT-IR spectral data of NH_2_-Phen/TiO_2_, NO_2_-Phen/TiO_2_, Phen/TiO_2_, and TiO_2_. The peaks at 589 cm^−1^ and 1638 cm^−1^ are attributed to the vibration of Ti–O–Ti (Xu et al., 2010) and stretching vibration of C=N of Phen (Li et al., 2016), respectively. After different groups were introduced into Phen/TiO_2_, NO_2_-Phen/TiO_2_, and NH_2_-Phen/TiO_2_, the infrared absorption peaks of these groups were also observed. The antisymmetric stretching vibration of −NO_2_ was observed at 1,584 cm^−1^; the ν(N-H) bands of −NH_2_ were observed at 1,416 and 3,411 cm^−1^ (Li et al., 2016). These results indicate that the corresponding derivatives of Phen are successfully combined with TiO_2_. As noted in the FT-IR spectrum, the C=C stretching band at 1,520 cm^−1^ of Phen/TiO_2_ shifted to 1,462–1,470 cm^−1^ of NO_2_-Phen/TiO_2_ and NH_2_-Phen/TiO_2_, indicating that the −NO_2_ and −HN_2_ groups increase the conjugation length of Phen (Feng et al., 2005). Meanwhile, the peak of NH_2_-Phen/TiO_2_, NO_2_-Phen/TiO_2_, and Phen-TiO_2_ has redshifted compared with that of TiO_2_, which may be due to the strong interaction between TiO_2_ and the derivatives of Phen.

**FIGURE 5:**
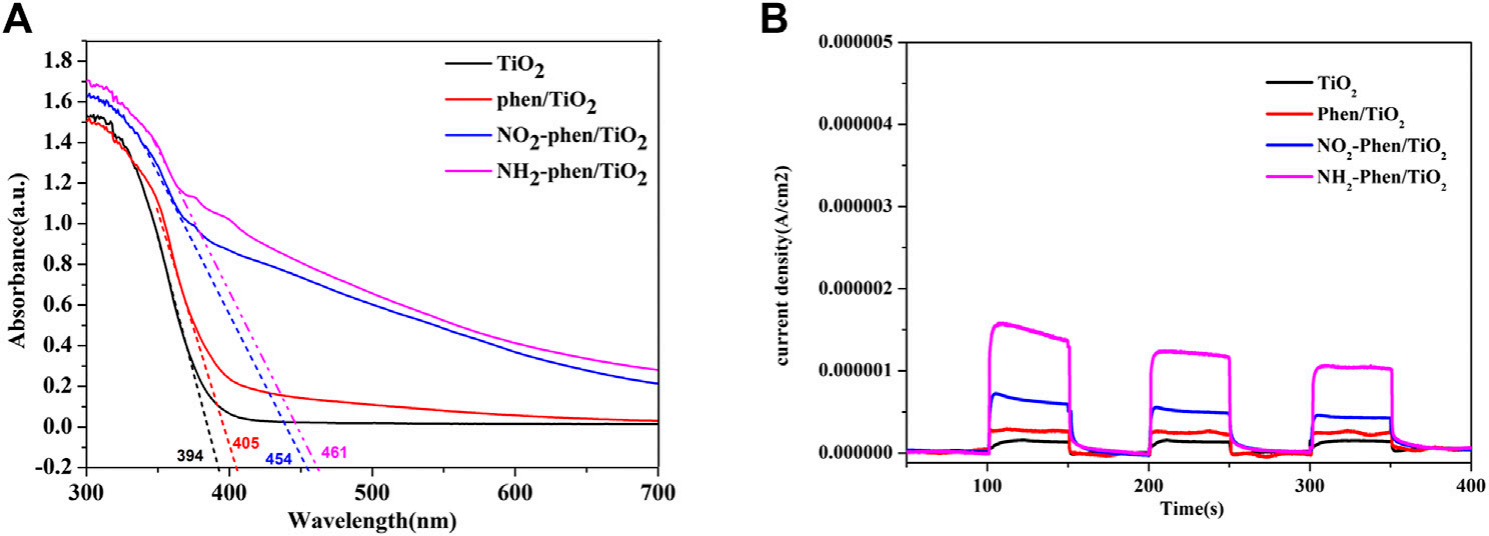
UV–Vis spectra **(A)** and photocurrent response **(B)** of NH_2_-Phen/TiO_2_, NO_2_-Phen/TiO_2_, Phen/TiO_2_, and TiO_2_.

Figure 5A shows the UV–Vis diffuse reflectance spectra (DRS) of NH_2_-Phen/TiO_2_, NO_2_-Phen/TiO_2_, Phen-TiO_2_, and bare TiO_2_. It can be seen from the figure that the absorption edges of all NH_2_-Phen/TiO_2_, NO_2_-Phen/TiO_2_, and Phen-TiO_2_ catalysts exhibit an obvious redshift to a higher wavelength, and the intensities are stronger in the visible range than that of TiO_2_. It can be indicated that the response range of TiO_2_ under visible light has been broadened after the modification of TiO_2_ by Phen and its derivatives. In addition, the redshift of the absorption edge indicates the decrease in band gap energy (Luo et al., 2013), and the band gap of all catalysts can be calculated as follows (Xu et al., 2018):

$$E_{g}={1240/\lambda }_{g},$$ (1)

where E_g_ and λ_g_ are the band gap energy and absorption edge of the photocatalyst, respectively. The absorption edge of TiO_2_, Phen/TiO_2_, NO_2_-Phen/TiO_2_, and NH_2_-Phen/TiO_2_ is approximately 394, 405, 454, and 461 nm, respectively. Consequently, the band gap energy (EG) of TiO_2_, Phen/TiO_2_, NO_2_-Phen/TiO_2_, and NH_2_-Phen/TiO_2_ is 3.15, 3.06, 2.73, and 2.69 eV, respectively. Thus, NH_2_-Phen/TiO_2_ has the largest redshift and the narrowest band gap energy, and the redshift and EG are in the order of NH_2_-Phen/TiO_2_ > NO_2_-Phen/TiO_2_ > Phen/TiO_2_, which is consistent with the result of the photocatalytic degradation. Based on this, we indicate that introducing a delocalized π-conjugated system with the amino group into TiO_2_ has a significant effect on the optical performance of the photocatalyst.

The photocurrent responses of the four catalysts under visible light irradiation are shown in Figure 5B. It is notable that the photocurrent intensity value of NH_2_-Phen/TiO_2_ after stabilization is about 1.0 μA, which is significantly higher than that of the other samples, indicating higher efficient charge-carrier separation at the interface of NH_2_-Phen/TiO_2_ (Jiang et al., 2018; Wu et al., 2018). The stronger photocurrent intensity indicates that the generation, separation, and transfer efficiency of NH_2_-Phen/TiO_2-_photogenerated electron–hole pairs are higher, and the recombination rate of electron–hole pairs is lower. At the same time, the photocurrent intensity value of NO_2_-Phen/TiO_2_, Phen/TiO_2_, and TiO_2_ is about 0.44, 0.28, and 0.14 μA, respectively. The results are in the order of NH_2_-Phen/TiO_2_ > NO_2_-Phen/TiO_2_ > Phen/TiO_2_, which is consistent with the results of the photocatalytic degradation and the DRS.

**FIGURE 6:**
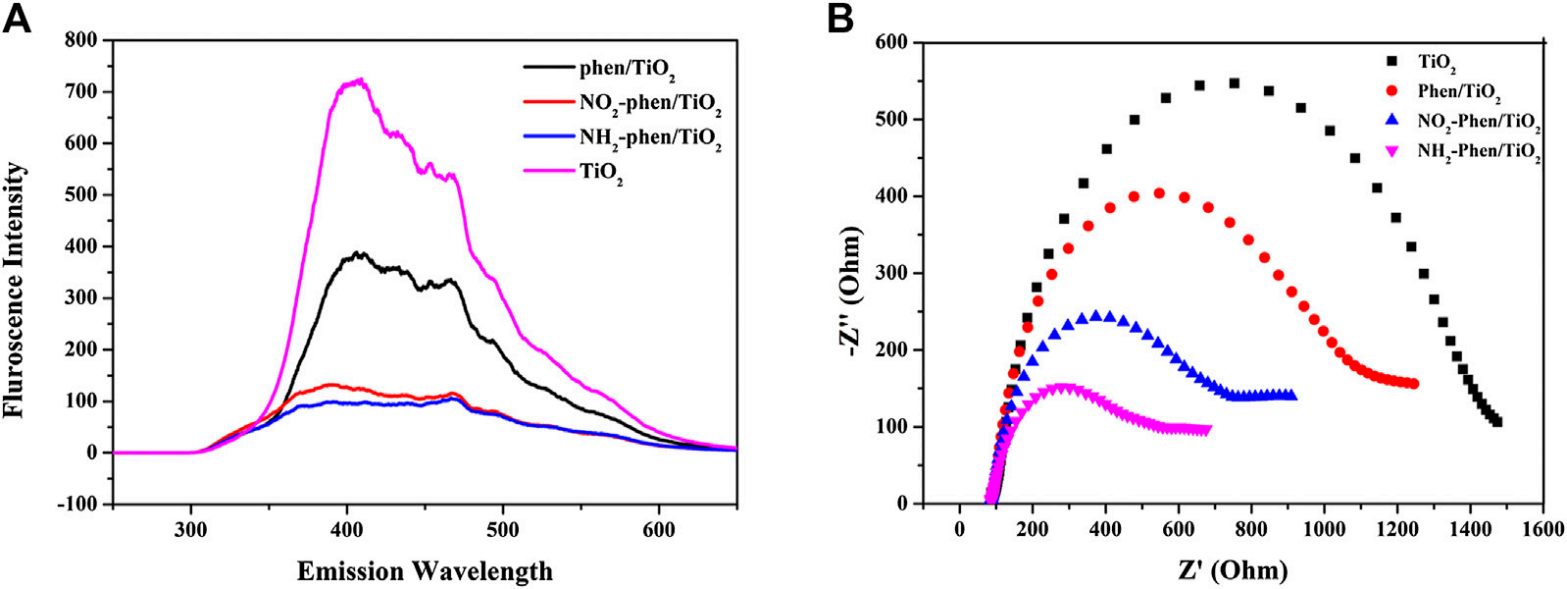
Photoluminescence spectra **(A)** and electrochemical impedance spectroscopy **(B)** of NH_2_-Phen/TiO_2_, NO_2_-Phen/TiO_2_, Phen/TiO_2_, and TiO_2_.

The separation of photoinduced electron–hole pairs is important for photocatalysis and can be explored from photoluminescence (PL) spectroscopy (Zhou et al., 2018). As shown in Figure 6A, the PL intensities of all Phen/TiO_2_, NO_2_-Phen/TiO_2_, and NH_2_-Phen/TiO_2_ are lower that of bare TiO_2_, indicating that the modification of Phen and its derivatives can suppress the recombination of electron–hole pairs, because higher PL intensity implies more drastic recombination of charge carriers, which favor the photocatalytic reactions. Comparing the PL intensity of the photocatalysts in Figure 6A, it is not difficult to speculate that the recombination rate of electron–hole pairs is in the order of Phen/TiO_2_ > NO_2_-Phen/TiO_2_ > NH_2_-Phen/TiO_2_, which indicates that a delocalized π-conjugated system with the amino group is more conducive to suppress the recombination of electron–hole pairs. Subsequently, the electrochemical impedance spectra (EIS) are obtained to investigate the charge transport properties of Phen/TiO_2_, NO_2_-Phen/TiO_2_, NH_2_-Phen/TiO_2_, and bare TiO_2_. As shown in Figure 6B, NH_2_-Phen/TiO_2_ has the smallest arc radius and Phen/TiO_2_ has the largest arc radius except bare TiO_2_ (Dai et al., 2015). The smallest arc radius implies the fastest interfacial charge-transfer properties, which facilitates subsequent photocatalytic reactions. The photocatalytic performance speculated by PL and EIS is in the order of NH_2_-Phen/TiO_2_ > NO_2_-Phen/TiO_2_ > Phen/TiO_2_, which is consistent with the result of the photocatalytic degradation experiment.

**FIGURE 7:**
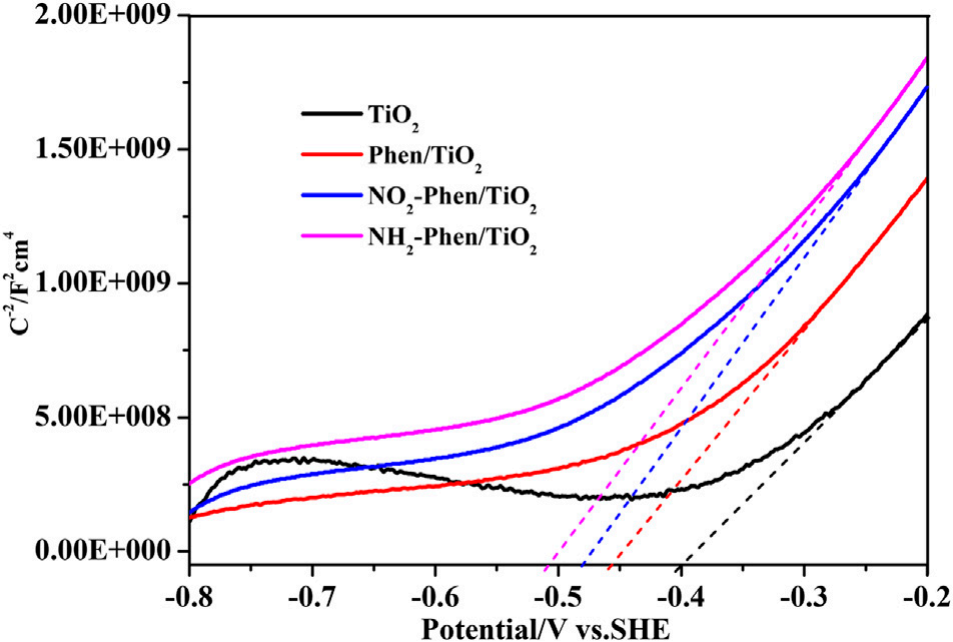
Mott–Schottky plots of TiO_2_, Phen/TiO_2_, NO_2_-Phen/TiO_2_, and NH_2_-Phen/TiO_2_.

Figure 7 shows the Mott–Schottky plots of bare TiO_2_ and Phen/TiO_2_, NO_2_-Phen/TiO_2_, and NH_2_-Phen/TiO_2_, and all the catalysts have positive slopes of Mott–Schottky plots, indicating that these catalysts are n-type semiconductors (Zhang and Cheng, 2009). According to the tangent line of the Mott–Schottky curve (Figure 7), the calculated flat-band potential energy V_fb_ of NH_2_-Phen/TiO_2_, NO_2_-Phen/TiO_2_, Phen/TiO_2_, and TiO_2_ is −0.51, −0.48, −0.46, and −0.40 eV vs. SCE and −0.27, −0.25, −0.23, and −0.21 eV vs. SHE, respectively. In general, as an n-type semiconductor, the flat-band potential energy is equal to its Fermi level, while the conduction band (CB) potential is approximately 0.2 eV less than its Fermi level (Ishikawa et al., 2002; Zhou et al., 2011). Thus, E_CB_ of NH_2_-Phen/TiO_2_, NO_2_-Phen/TiO_2_, Phen/TiO_2_, and TiO_2_ is −0.47, −0.45, −0.43, and −0.41 eV vs. SHE, respectively. According to the E_G_ value estimated by DRS and the empirical formula (E_G_ = E_VB_ − E_CB_), the corresponding valence band (VB) potentials can be calculated as 2.22, 2.28, 2.63, and 2.74 eV, respectively.

**FIGURE 8:**
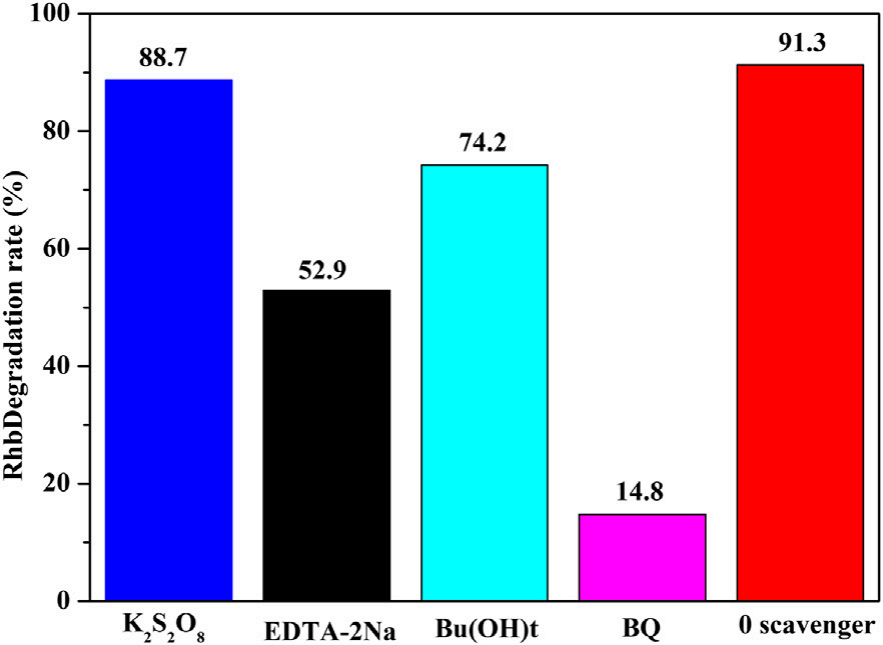
Photocatalytic degradation of MO by NH_2_-Phen/TiO_2_ in the presence of different capture agents under visible light (70 mg catalysts and 70 ml MO solution with a concentration of 10 mg/L).

To understand the possible mechanism for the improved photocatalytic activity of NH_2_-Phen/TiO_2_, trapping experiments were performed to identify the active species for the photodegradation (Zou et al., 2016; Dong et al., 2017). There are four active species (e^−^, h^+^, •OH radicals, and •O_2_^−^ radicals that can be captured by t-BuOH, K_2_S_2_O_8_, DETA-2Na, and BQ, respectively) that play important roles in the photocatalytic reaction process (Jiang et al., 2018). As seen in Figure 8, the photocatalytic degradation rate of MO under visible light without any trapping agent is 91.3%, while the degradation efficiency of MO by adding t-BuOH, K_2_S_2_O_8_, DETA-2Na, and BQ is 74.2, 88.7, 52.9, and 14.8%, respectively. These results indicate that •O_2_^−^ is the main active species responsible for the degradation of MO and h^+^ is the secondary active species. According to the results of the Mott–Schottky analysis, the E_CB_ and valence potential of NH_2_-Phen/TiO_2_ are approximately −0.47 eV vs. SHE and 2.22 eV vs. SHE, which are lower than the reduction potential of O_2_/•O_2_^−^ (−0.33 V) and •OH/OH (2.38 V) (Shao et al., 2015). Therefore, when NH_2_-Phen/TiO_2_ is irradiated by visible light during the period of photodegradation of MO, O_2_ is reduced to •O_2_^−^ by electrons, while OH^−^ cannot be oxidized to •OH by holes.

**FIGURE 9:**
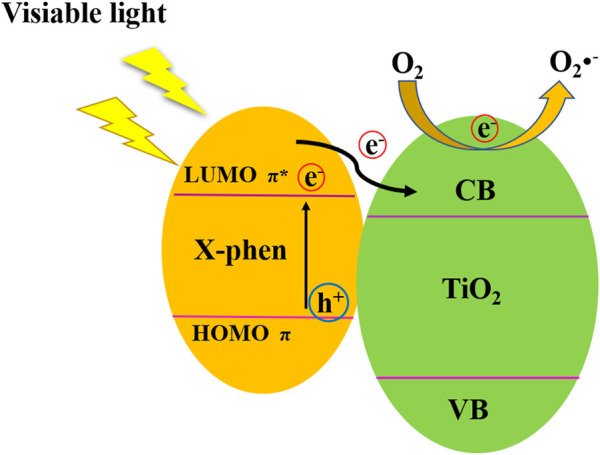
Possible visible light photocatalytic mechanism of NH_2_-Phen/TiO_2_, NO_2_-Phen/TiO_2_, and Phen/TiO_2_ nanoparticles.

On the basis of the above results, the possible mechanism of NH_2_-Phen/TiO_2_, NO_2_-Phen/TiO_2_, and Phen/TiO_2_ photocatalytic degradation of methyl orange (MO) is proposed in Figure 9. Phen and its derivatives can greatly promote the dispersion of the nanoparticles, resulting in more active sites to be exposed. Furthermore, Phen and its derivatives can act as sensitizers to enhance visible light absorption. Under the irradiation of visible light, the delocalized π-conjugated Phen and its derivatives on the surface of NH_2_-Phen/TiO_2_, NO_2_-Phen/TiO_2_, and Phen/TiO_2_ nanocomposites can easily absorb visible light to induce a π–π* transition state and then generate electron–hole pairs. The excited electrons in the LUMO of Phen and its derivatives will be easily injected into the conduction band of TiO_2_. A fast photoinduced electron-transfer reaction takes place between the conjugated organic system (electron donor) and TiO_2_ (electron acceptor), which effective suppresses the recombination of the photogenerated electron–hole pairs (Luo et al., 2012). Consequently, electrons would be captured by H_2_O or oxygen adsorbed on the surface of the photocatalysts to produce •O_2_^−^, which involves in the degradation of MO along with h^+^, which conduce to improve the visible light photocatalytic activity of NH_2_-Phen/TiO_2_, NO_2_-Phen/TiO_2_, and Phen/TiO_2_ nanocomposites.

## Conclusion

Novel TiO_2_-based hybrid photocatalysts containing different delocalized π-conjugated systems (NH_2_-Phen/TiO_2_, NO_2_-Phen/TiO_2_, and Phen/TiO_2_) and TiO_2_ were successfully synthesized *via* the hydrothermal method. Regardless of DRS, photocurrent response, PL, EIS, and photocatalytic degradation of MO, the results demonstrate that delocalized π-conjugated Phen and its derivatives greatly broadened the range of light absorption and effectively promoted the transfer of photogenerated electron–hole pairs; thus, NH_2_-Phen/TiO_2_, NO_2_-Phen/TiO_2_, and Phen/TiO_2_ exhibited much higher visible light photocatalytic activity than TiO_2_. NH_2_-Phen/TiO_2_ showed the highest photocatalytic performance in the degradation of MO under visible light irradiation, which indicated that introducing a delocalized π-conjugated system with the amino group into TiO_2_ was more favorable in improving the photocatalytic activity. This study provides a guide for the synthesis of highly efficient TiO_2_-based catalysts at the molecular level.

## Data Availability

The original contributions presented in the study are included in the article/Supplementary Material; further inquiries can be directed to the corresponding authors.
