# Supplementary material for: Effects of Different Delocalized π-Conjugated Systems Towards the TiO2-Based Hybrid Photocatalysts
Source: Front Chem. 2021 Jul 27;9:700380. doi: 10.3389/fchem.2021.700380 (PMC8353090; doi:10.3389/fchem.2021.700380)
Supplement: Supplementary file 1 [file DataSheet1.docx]

Effects of different delocalized π conjugated systems towards the TiO_2_ based hybrid photocatalysts

Weibo Zhang^1^, Pinghua Chen^2,3^, Jun Liu^2,3^, NanNan Huang^4^, Chenglian Feng^4^, Daishe Wu^1*^, Yingchen Bai^4*^

^1^Key Laboratory of Poyang Lake Environment and Resource Utilization, Ministry of Education, School of Resources Environmental & Chemical Engineering, Nanchang University, Nanchang 330031, China

^2^Key Laboratory of Jiangxi Province for Persistent Pollutants Control and Resources Recycle, Nanchang 330063, Jiangxi, China

^3^College of Environmental and Chemical Engineering, Nanchang Hangkong University, Nanchang 330063, Jiangxi, China

^4^State Key Laboratory of Environmental Criteria and Risk Assessment, Chinese Research Academy of Environmental Sciences, Beijing 100012, China





Fig. S1 Adsorption capacities in dark of TiO_2_ and the modified TiO_2_ catalysts towards MO (the initial concentration of MO is 10 mg/L; the catalysts concentration is 1 g/L)
